# Monitoring of plant-induced electrical signal of pepper plants (*Capsicum annuum* L.) according to urea fertilizer application

**DOI:** 10.1038/s41598-022-26687-w

**Published:** 2023-01-06

**Authors:** Han Na Kim, Yeong Ju Seok, Gyung Min Park, Govind Vyavahare, Jin Hee Park

**Affiliations:** Department of Environmental and Biological Chemistry, Chungbuk National University, 1 Chungdae-ro, 28644, Cheongju, Seowon-gu Republic of Korea

**Keywords:** Plant sciences, Environmental sciences

## Abstract

Plant-induced electrical signals (PIES) can be non-destructively monitored by inserting electrodes into plant stems, which reflect plant nutrient and water uptake. The main objective of this study was to evaluate the growth of pepper plants with different urea applications (low fertilizer: N0, Control: N1, and high fertilizer: N2) in soil by monitoring PIES. The PIES value was found to be low in the low urea treatment group while the two times higher urea applied pepper had the highest PIES value. The nutritional content of the stem, leaves and soil did not correlate with PIES because of dilution effect by high biomass with high urea application, but principal component analysis showed that the PIES was positively associated with pepper biomass and soil EC. The high fertilizer did not affect chlorophyll and proline contents in pepper leaves. The assessment of plant growth by PIES has advantages because non-destructive, real time and remote monitoring is possible. Therefore, PIES monitoring of different plants grown under various cultivation environments is useful method to evaluate plant activity and growth.

## Introduction

It is important to precisely manage soil nutrients in agriculture to maintain crop productivity and reduce nutrient pollution caused by applying fertilizers while considering the growing environment, such as crops, climate and water^1^. However, higher or lower fertilizer produces nutritional imbalances in the soil. Nutrient insufficiency leads to poor crop development, augmented plant stress, and also reduces plant productivity^2^. If excessive nutrients are presented in the soil, it leads to the deterioration of physicochemical characteristics of the soil and subsequently lower the crop productivity^3^. Lack of nutrients in the soil can also cause the plant's leaves to turn yellow or perish^4^. The environment is also affected by nutrient imbalances in the soil^5^. Nitrogen and phosphorus promote plant development, but they are stimulating eutrophication, water pollution, and greenhouse gas emissions^6,7^. The mineral nutrients, N, P and K are known to affect growth and yield of the capsicums^8^. Therefore, soil nutrient management is an influential task since it can reduce pollution while also augmenting crop growth and yield^9^.

The main reason for over-fertilizing the soil is that it is difficult to assess the growth of plants^10^. In addition, it is strenuous to evaluate when plant growth is delayed or there is an anomaly since symptoms may not manifest immediately, making it more difficult to apply nutrients based on the condition of the plant’s health^11^. Plant stress or growth can be evaluated by measuring the chlorophyll and proline levels of plants, but this method is time-consuming and destructive^12^. Instead, a sensor or machine that can monitor plant stress and growth in real-time can be used to manage nutrient application and has recently been investigated^13,14^.

Plant-induced electrical signals (PIES) use electrodes on both sides of the plant stem to measure the internal resistance, convert it to electrical conductivity, and reflect the absorption of water in the stem^15^. Therefore, PIES can be used to monitor plant growth and stress in different cultivation environments. PIES is predominantly associated to plant growth and used to evaluate effect of environments such as temperature, humidity, CO_2_, and light on plant growth. Previous research has demonstrated that it can be used to evaluate the temperature-dependent growth of broccoli (*Brassica oleracea* var. *italica*) and pepper (*Capsicum annuum* L.)^16,17^. Measurements of the PIES can also be used to evaluate ion transport in relation to urea applied to the soil where broccoli is cultivated^18^. However, it's unclear how different soil nutrient content affects the physiological responses of various plants and how PIES responds. Therefore, in order to develop an optimal nutrient application level based on plant growth using PIES, it is required to evaluate the relation between nutrient content and plant physiological response by monitoring the growth of various plants by PIES. Urea is the most often used nitrogen fertilizer in agriculture. Therefore, the main goal of this study was to evaluate how urea administration affected changes in mineral nutrient status in pepper plants and their growth using PIES.

## Materials and methods

### Pepper growth and treatment of urea

Pepper (*Capsicum annuum* L.) seedlings were cultivated in horticultural soil for one month before being transplanted to sandy loam soil at 4 kg per pot. The characteristics of soils used were provided in Supplementary Table 1. The use of plants in the present study complied with international, national and/or institutional guidelines. Three treatment groups were evaluated how N fertilizer application affected pepper growth in the same environment. The total amount of N–P–K for control was 19.0–11.2–15.0 kg/10 a, respectively, which was applied based on the standard pepper fertilization amount^19^. Micronutrients were not supplied because microelements can be supplied by the soil. The same amount of basal fertilizer was given to each plant except the low fertilizer treatment, and different amount of additional nitrogen fertilizer was added for different treatment. The low fertilizer (N0) did not receive any fertilizer, and the control (N1) was treated with urea fertilizer with an appropriate nitrogen amount of 10.3 kg/10 a. The high fertilizer (N2) was treated with a quantity of urea 20.6 kg/10 a, which was 2 times that of the control (N1) treatment group. After harvest of pepper plants, plant height, stem diameter, and fresh and dry weight were measured. Stems were used for sap extraction, leaves were washed with distilled water, and 10 g of leaves were frozen at −40 ℃ until further usage for chlorophyll and proline analysis. The rest was oven-dried at 60 °C and utilized for elemental analysis.

### Field experiments

Basal fertilizer including compost was supplied in all groups. The amount of additional fertilizer in the low fertilizer (N0) and control (N1) treatment groups was the same as in the greenhouse experiment, and the high fertilizer (N1.5) treatment group was applied with 15.5 kg/10 a of nitrogen, which was 1.5 times of the amount of urea in control. Pepper was cultivated for 14 weeks and after harvest analytical samples were prepared same as in the greenhouse experiments.

### Monitoring of PIES

PIES was used to observe the response of pepper plants to various levels of nitrogen fertilizer application during the growing season. Three stainless steel needle electrodes were inserted into the pepper stem at a distance of five centimeters from the ground and five millimeters on each side. Schematic diagram and photo of the sensor were provided in Supplementary Fig. 1. The stem electrical resistance was measured using Junsmeter II (Prumbio, Korea) and the electrical resistance was converted to electrical conductivity using Eq. (1)^15^.

1 $$\mathrm{PIES}=\mathrm{k}\frac{1}{R}\times \frac{D-L}{L\times d}$$

where *R* is the resistance of the pepper stem (Ω), D is the diameter of the pepper stem (mm), L is the length of the inserted needle (mm), d is the diameter of the needle (mm), and k is a constant.

### Nutrient analysis of plant and soil

Following the plant harvest, the fresh weight of the five-centimeter stem was measured at the point where the PIES measurement electrodes were introduced. Pepper stem from the greenhouse experiment was placed in a 15 mL conical tube with 10 mL distilled water, and stem from the field experiments was placed in a 50 mL conical tube with 30 mL distilled water and extracted by stirring for 24 h^20^. The pH and EC of the extracted solution were also determined, and the solution was filtered with a syringe filter before being analyzed with ICP-OES (Avio 500, Perkin Elmer). Furthermore, a UV–VIS spectrophotometer (Orion AquaMate 7000, Thermo-Fisher Scientific) was used to evaluate ammonium nitrogen content in stem extract using the indophenol method^21^.

The elemental content of pepper leaves in each treatment group was measured. The plant leaf sample was crushed in a mortar and pestle, and 0.1 g of the sample was placed in a 100 mL conical flask, along with 5 mL of nitric acid, and digested at 140 °C until the volume was decreased to about 1 mL. The digested sample was diluted to 50 mL with distilled water and the element contents in the solution were determined using ICP-OES.

When the pepper plants were harvested, a soil sample was collected from the root zone, dried at room temperature, and sieved to 2 mm and used for analysis. The soil sample (5 g) was added in a 50 mL conical tube with 25 mL distilled water, and the mixture was agitated at 180 rpm for 30 min. The pH and EC of the extracted solution were measured according to the methods of Rhoades^22^. A solution extracted at a ratio of 1:10 using 1 N C_2_H_7_NO_2_ was pretreated in the same way and the exchangeable cation content was analyzed using ICP-OES^23^.

### Analysis of Chlorophyll and proline content of leaves

Frozen pepper leaves (0.5 g) were crushed in 25 mL 80% acetone and the mixture was kept in the dark room at 4 °C for 24 h. The contents of chlorophyll *a* and chlorophyll *b* were calculated using absorbance measured at 645 and 665 nm, respectively^24^.

To extract proline from pepper leaves, 0.5 g of frozen leaves were combined with 10 mL of 3% sulfosalicylic acid in a 50 mL conical tube and sonicated for 30 min. The extracted solution was centrifuged for 10 min at 25 °C and 4000 rpm before being filtered through a 0.45 μm syringe filter. Further, the acid-ninhydrin reagent (2 mL) and glacial acetic acid (2 mL) were added to 2 mL of the filtrate, and the mixture was heated at 100 °C for 1 h^25^. After cooling, 4 mL of toluene was added to separate the chromophore, and absorbance was measured at 520 nm with toluene as a blank.

### Statistical analysis

Statistical analysis of the data was performed using SPSS software (IBM, Armonk, NY, USA). The PIES data were presented as an average value of triplicates. Growth, elemental content, chlorophyll, and proline content were expressed as mean and standard deviation. One-way ANOVA was adopted to evaluate the differences in between the treatment groups. Post hoc analysis was performed with Duncan's multi-range test at *p* value < 0.05. Principal component analysis (PCA) (Xlstat, Addinsoft) was employed to evaluate relationship among PIES, elemental contents of stem and soil and chlorophyll and proline contents.

## Results and discussion

### PIES by different urea fertilizer applications

**Figure 1:**
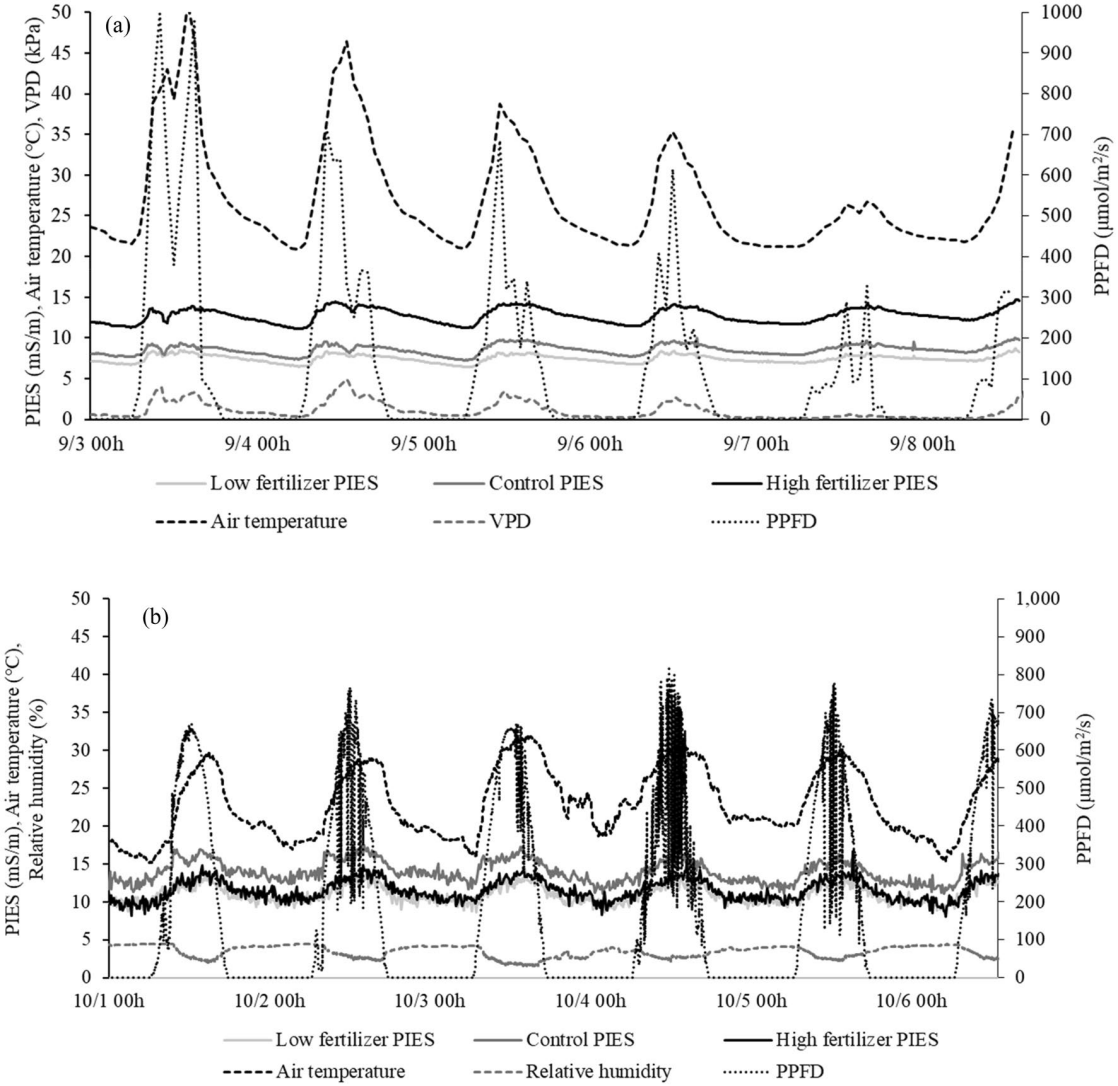
Plant induced electrical signal (PIES) of pepper plants according to different urea fertilizer applications in a greenhouse (**a**) and in field (**b**).

In all treatment groups of greenhouse and field experiment, the pattern of PIES was found to be the similar to air temperature pattern with a different peak intensity on daily basis (Fig. 1). Park et al.^15^ and Cha et al.^16^ stated that the pattern of PIES showed a diurnal cycle similar to air temperature and PPFD, which was related to water and nutrient uptake of the plant. During the day period, evaporation and transpiration rate of the plant was high owing to an increase in the movement of water and ions in a plant's vessel^26^. PIES has a pattern that increases during the day and decreases at night because plants are more active at elevated temperatures with PPFD, which increases their ability to absorb nutrient and water^27^. Therefore, PIES can be used as an indicator to evaluate the nutrient absorption capacity, physiological activity, and growth status of plants under various environmental conditions^15^.

In the greenhouse experiment, the PIES for urea (N) application was measured as follows; N0 < N1 < N2 (Fig. 1a). The values of PIES increased as the urea application increased indicating that greater PIES values for the application of a higher dose of urea (N2). The high PIES value can be associated with better plant growth because growth index such as shoot fresh and dry biomass was in the order of N0 < N1 < N2 treated groups (Table 1). Although stem diameter and shoot height were not significantly different among treated groups, they were also in the order of N0 < N1 < N2 indicating that higher urea application resulted in higher plant growth (Table 1). Nitrogen deficiency inhibits protein synthesis, impeding plant growth, but excessive amounts of N application can be hazardous to plant roots and young plants^28,29^. However, in this experiment, two times higher dose of N fertilizer promoted the growth of pepper plants.

**Table 1:** Analysis of various growth parameters of the pepper plants according to different urea applications in a greenhouse (a) and in field (b). Significant differences among different treatment groups were marked with different letters (*p* < 0.05).

	Stem diameter (cm)	Shoot height (cm)	Shoot fresh weight (g)	Shoot dry weight (g)	Root fresh weight (g)	Root dry weight (g)
**(a)**						
Low fertilizer (N0)	6.78 ± 0.46 a	58.2 ± 6.9 a	30.4 ± 4.6 c	5.26 ± 0.53 c	7.18 ± 0.48 b	1.37 ± 0.12 b
Control (N1)	7.09 ± 0.97 a	65.3 ± 11.5 a	79.5 ± 8.8 b	12.78 ± 2.91 b	11.35 ± 1.38 a	2.15 ± 0.22 a
High fertilizer (N2)	7.69 ± 0.63 a	67.7 ± 9.8 a	133.4 ± 11.0 a	24.01 ± 1.95 a	11.52 ± 2.62 a	2.10 ± 0.51 a

	Stem diameter (mm)	Shoot height (cm)	Shoot fresh weight (g)	Fresh weight per pepper fruit (g)	Dry weight per pepper fruit (g)	
**(b)**						
Low fertilizer (N0)	21.6 ± 0.4 a	133.5 ± 15.6 a	520 ± 115 b	1.6 ± 0.3 a	0.6 ± 0.3 a	
Control (N1)	21.7 ± 0.1 a	141.5 ± 12.4 a	661 ± 88 ab	1.5 ± 0.6 a	0.6 ± 0.6 a	
High fertilizer (N1.5)	22.2 ± 2.5 a	151.6 ± 0.6 a	799 ± 136 a	1.1 ± 0.4 a	0.4 ± 0.1 a	

PIES for different urea applications was measured in the field experiment as follows: N0 < N2 < N1, but PIES was not much different among different urea treatment groups (Fig. 1b). When the growth index of pepper plants was examined, no significant differences were found in different urea applications in all groups (Table 1). In this study, basal fertilizer including compost was supplied in all groups and the amount of additional urea was different. Therefore, this result is believed to have occurred because the difference in fertilizer throughput did not enough to affect the growth of pepper plants. In the field experiment, high fertilizer treatment was applied with less amount of urea (1.5 times) compared to greenhouse experiment (2 times) to prevent contamination of the surrounding environment. Furthermore, greenhouses (pots) provide controlled environment in which plants can grow well, while also increasing the efficiency of fertilizer recovery^30^. However, plants grown in fields were exposed to external environmental factors such as wind, rainfall, and temperature, which increases leaching and evaporation, resulting in lower fertilizer recovery^31^. Therefore, in this experiment, plants grown in the field might be less affected by fertilizers due to external environmental factors, yielding results that differ from those obtained in the greenhouse^32^.

### Nutrient uptake according to urea application and its relation to PIES

Daytime PIES values were averaged to compare PIES values with other destructively measured parameters. Destructively measured parameters mean parameters measured after harvest of plant samples and digestion or extraction of plant tissues, which include nutrient, chlorophyll and proline contents in pepper plants. The PIES was not related with the ion content of the stem extract. The PIES was the highest in high fertilizer treated group and element concentrations such as K, Mg, P and S were the lowest, which might be attributed to dilution effect because the nutrient content of the stem extract was calculated as the nutrient content per dry weight of the stem (Table 2). Especially, K concentration was correlated (R^2^ = 0.5) with fresh stem biomass used for the extraction. Han et al.^33^ also showed that different fertilizer treatments did not significantly affect nutrient concentrations in stems, but it significantly increased dry weight of stems. Stem nutrient concentrations other than applied nutrient was not good indicators of nutritional status because of dilution effect^34^. Nitrogen concentrations were not different because ammonium absorbed by plants is rapidly assimilated into amide compounds to prevent toxic damage caused by an accumulation of NH_4_^+^^35^. The PIES can be related to the nutrient and water flow rather than nutrient concentrations in the stem. The element content of plant stem extract and amount of sap were related to the plant's nutritional absorption ability because nutrients were available to plants in the form of water-soluble ions and they passed through vessels with sap^36^. In the field experiment, there was no significant difference in the nutrient content of the stem extract in all applications. Nutrient concentrations in pepper leaves grown in pot of greenhouse were highest in high fertilizer treated groups. However, significant differences were not found in pepper plants grown in field (Table 3). This result is consistent with the fact that there was no substantial difference in the growth of pepper plants owing to a lower or higher dose of fertilizer application.

**Table 2:** PIES and nutrient concentrations of pepper stem extract (mg/kg).

	Greenhouse			Field		
	Low fertilizer (N0)	Control (N1)	High fertilizer (N2)	Low fertilizer (N0)	Control (N1)	High fertilizer (N1.5)
PIES (mS/m)	8.02 ± 2.01 b	9.63 ± 1.56 b	14.10 ± 2.65 a	12.83 ± 2.02 a	16.68 ± 3.29 a	13.71 ± 2.27 a
Ca	73.29 ± 15.21 a	72.85 ± 4.59 a	59.60 ± 7.54 a	41.94 ± 2.46 a	38.52 ± 3.56 a	35.69 ± 1.41 a
K	522.1 ± 10.6 ab	582.7 ± 14.3 a	423.2 ± 93.6 b	474.8 ± 46.8 a	618.8 ± 19.6 a	646.8 ± 211.5 a
Mg	45.54 ± 7.72 a	43.25 ± 4.27 ab	33.75 ± 1.46 b	44.38 ± 1.85 a	39.07 ± 3.88 a	44.48 ± 3.84 a
P	22.93 ± 1.47 a	7.00 ± 1.41 b	4.87 ± 4.96 b	7.60 ± 5.76 a	6.00 ± 4.83 a	9.96 ± 3.02 a
S	116.7 ± 15.4 a	78.71 ± 3.57 b	55.31 ± 9.44 c	14.52 ± 2.50 a	16.83 ± 1.77 a	15.60 ± 3.47 a
NH_4_^+^-N	8.96 ± 1.59 a	6.86 ± 1.22 a	6.84 ± 0.72 a	4.22 ± 0.47 a	7.65 ± 4.00 a	5.27 ± 1.63 a
NO_3_^–^N	–	–	–	0.09 ± 0.00 a	0.09 ± 0.03 a	0.08 ± 0.01 a
Stem extract EC (µS/cm)	210.0 ± 25.4 a	271.4 ± 48.9 a	226.1 ± 61.8 a	224.3 ± 35.9 a	270.7 ± 9.3 a	292.6 ± 67.3 a

Significant differences among different treatment groups were marked with different letters (*p* < 0.05).

**Table 3:** Nutrient concentrations of pepper leaves treated with different urea fertilizer applications (mg/kg).

	Greenhouse			Field		
	Low fertilizer (N0)	Control (N1)	High fertilizer (N2)	Low fertilizer (N0)	Control (N1)	High fertilizer (N1.5)
Ca	3322 ± 804 a	2262 ± 265 b	3908 ± 103 a	131,007 ± 89,664 a	128,320 ± 50,874 a	161,976 ± 11,280 a
K	2815 ± 744 ab	2319 ± 53 b	3514 ± 342 a	357,814 ± 36,879 a	408,398 ± 32,383 a	395,557 ± 34,324 a
Mg	795 ± 163 b	504 ± 74 b	1131 ± 181 a	67,180 ± 17,417 a	63,568 ± 10,085 a	79,108 ± 1259 a
P	383 ± 38 b	253 ± 10 b	1155 ± 218 a	50,127 ± 6894 a	49,167 ± 9417 a	60,556 ± 21,256 a
S	645 ± 67 b	577 ± 21 b	1076 ± 51 a	28,611 ± 7374 a	30,225 ± 1928 a	29,112 ± 7125 a

Significant differences among different treatment groups were marked with different letters (*p* < 0.05).

Water and ammonium acetate extractable nutrient concentrations of the soil were analyzed to evaluate nutrient availability after urea fertilization, but there was an insignificant difference in the elemental content except for K and P in greenhouse experiment (Table 4). Reduced K and P concentrations in control and high fertilizer amended pots can be attributed to higher biomass and uptake of these elements by plants. Although urea was fertilized at different amount for each treatment group, nitrogen concentrations in soil were not different because of nitrogen uptake by pepper plants and loss of nitrogen after transformation. Ren et al.^37^ also reported that nitrogen fertilization did not change total nitrogen content in soil due to changes in nitrogen fraction. Lower pH of control (N1) and high fertilizer (N2) treated soil compared to low fertilizer (N0) in greenhouse experiment also could be caused by higher root biomass (Tables 1, 4). In addition, inorganic fertilizer also reduced soil pH^38^.

**Table 4:** Water and ammonium extractable nutrient concentrations of soils treated with different urea fertilizer (mg/kg).

Nutrients	Extractant	Greenhouse			Field		
		Low fertilizer (N0)	Control (N1)	High fertilizer (N2)	Low fertilizer (N0)	Control (N1)	High fertilizer (N1.5)
Ca	H_2_O	20.22 ± 3.64 a	28.56 ± 6.43 a	17.30 ± 15.70 a	8.69 ± 2.73 a	5.02 ± 3.78 a	4.53 ± 1.73 a
	NH_4_OAC	158.0 ± 4.8 a	160.5 ± 13.5 a	156.5 ± 4.5 a	648.3 ± 79.3 a	601.5 ± 34.7 a	620.6 ± 182.5 a
K	H_2_O	10.24 ± 1.14 a	9.97 ± 2.44 ab	5.19 ± 4.34 b	44.28 ± 30.02 a	33.66 ± 12.82 a	20.35 ± 17.30 a
	NH_4_OAC	17.77 ± 1.45 a	14.45 ± 1.63 b	13.26 ± 0.53 b	326.7 ± 186.2 a	319.6 ± 117.3 a	221.9 ± 112.8 a
Mg	H_2_O	3.03 ± 0.65 a	4.74 ± 1.75 a	2.40 ± 2.17 a	5.37 ± 2.25 a	2.99 ± 2.19 a	2.29 ± 1.13 a
	NH_4_OAC	14.07 ± 0.41 a	14.55 ± 1.24 a	14.16 ± 0.66 a	227.0 ± 14.4 a	213.6 ± 16.1 a	173.5 ± 22.0 b
P	H_2_O	2.28 ± 0.14 a	2.12 ± 0.25 a	1.03 ± 0.86 b	0.65 ± 0.60 a	0.33 ± 0.07 a	0.24 ± 0.05 a
S	H_2_O	12.07 ± 1.39 a	14.51 ± 2.55 a	11.19 ± 9.23 a	10.72 ± 4.89 a	9.09 ± 3.35 a	8.57 ± 3.33 a
NH_4_^+^-N	KCl	12.71 ± 0.51 a	12.54 ± 0.51 a	14.52 ± 3.13 a	3.71 ± 1.88 a	1.45 ± 0.81 a	2.38 ± 0.49 a
pH		7.42 ± 0.12 a	7.15 ± 0.11 b	7.04 ± 0.07 b	6.25 ± 0.78 a	5.66 ± 0.40 a	5.28 ± 0.03 a
EC (µS/cm)		53.55 ± 7.56 b	79.94 ± 10.27 a	84.27 ± 11.74 a	63.51 ± 0.78 a	52.97 ± 0.40 a	29.93 ± 0.03 b

Significant differences among different treatment groups were marked with different letters (*p* < 0.05).

### Effect of urea application on the chlorophyll and proline content

**Figure 2:**
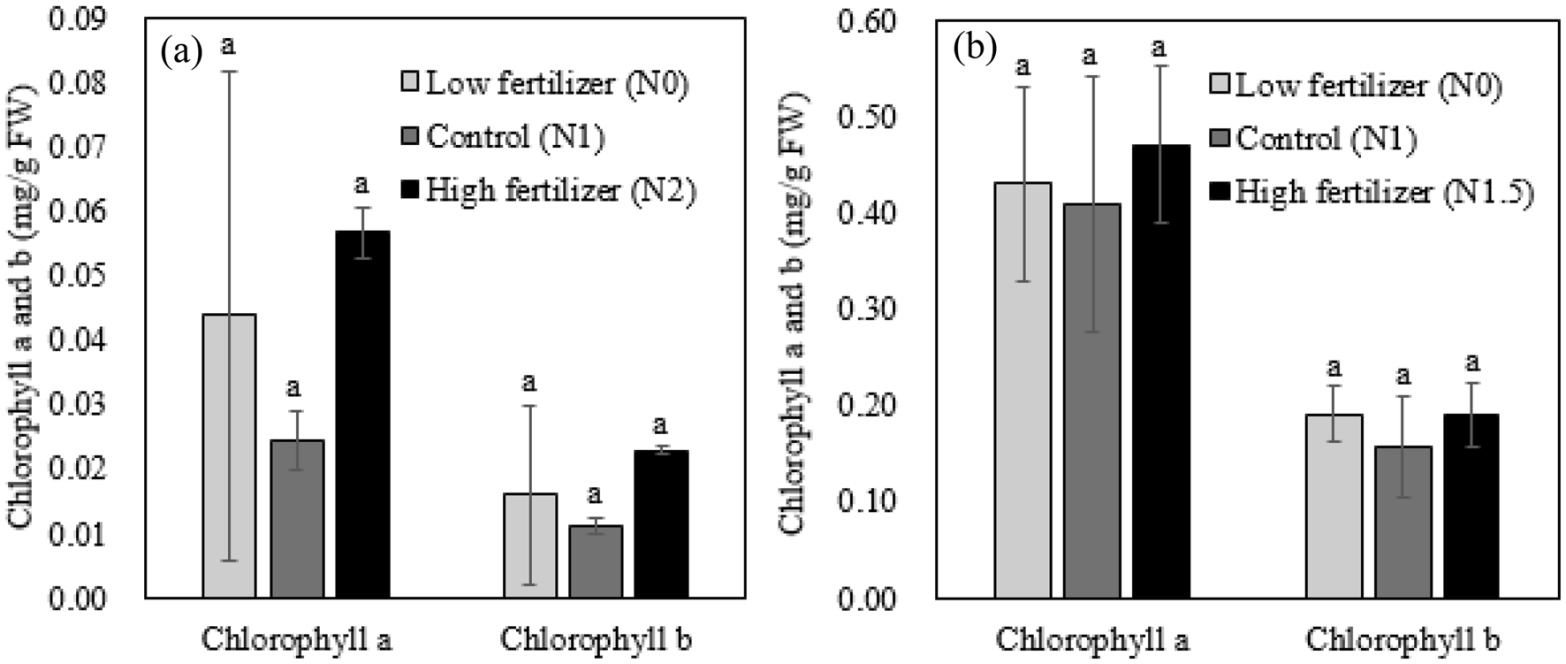
Chlorophyll content of pepper leaves treated with different urea fertilizer applications; greenhouse experiments (**a**) and field experiments (**b**).

Chlorophyll is associated with photosynthesis, and plants synthesize proline to prevent cell damage and resist environmental stress, so it can be used as a growth indicator^39^. In both experiments, the contents of chlorophyll a and b were slightly elevated in high urea treated groups, but significant differences were not found (Fig. 2). Generally, nitrogen influences thylakoid synthesis through the Calvin cycle resulting in an increase in the chlorophyll content^40^. However, applied urea levels were not different to induce differences in chlorophyll content in this study. Hokmalipour and Darbandi^41^ reported that two times of nitrogen fertilizer application did not increased chlorophyll content as measured by SPAD, but three times of nitrogen fertilizer significantly increased chlorophyll content.

**Figure 3:**
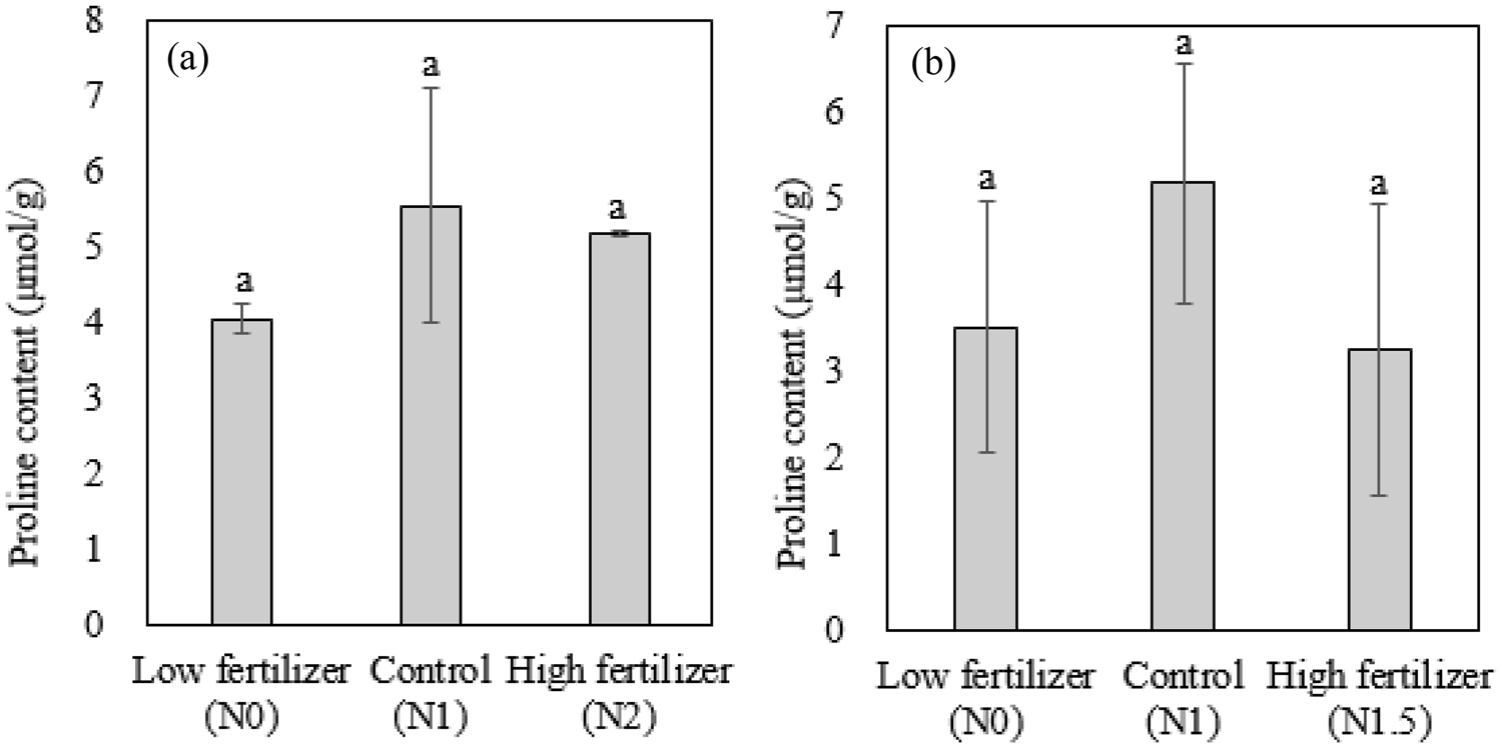
Proline content of pepper leaves treated with different urea fertilizer applications; greenhouse experiments (**a**) and field experiments (**b**).

Proline contents were not significantly different among different treatment groups indicating that plants were not stressed (Fig. 3). Cha et al.^16^ reported that proline content tended to increase under salt stress. As pepper biomass increased under high fertilizer application and proline content was not high, two times higher urea application was beneficial for pepper growth in this study.

### Relationship of growth parameters of pepper with PIES

**Figure 4:**
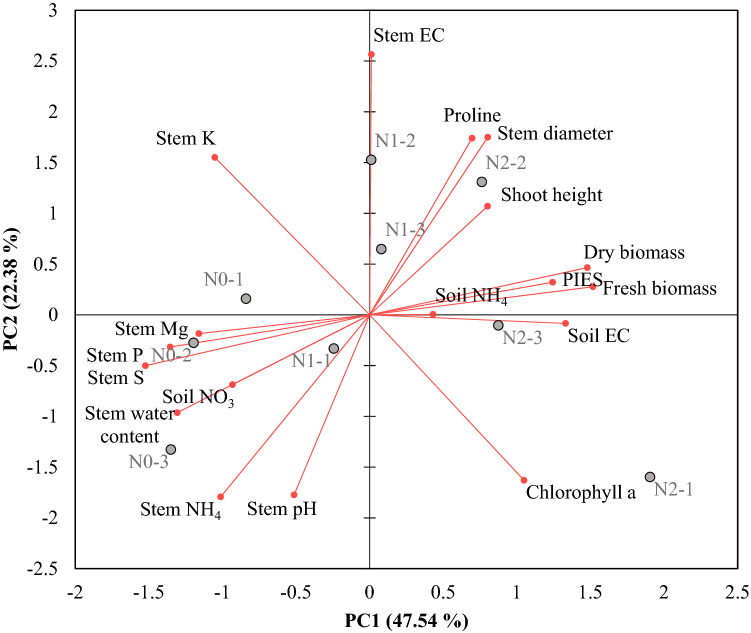
Biplot of PC1 and PC2 for growth parameters of pepper plants grown in different amount of urea applied soils (low fertilizer: N0, control: N1, high fertilizer: N2, numbers after dash indicate replicates).

Principal component analysis (PCA) was conducted to understand the association of destructively measured growth parameters of pepper and monitored PIES. The PCA was conducted for pepper plants grown in green house because pepper plants in field did not show significant differences with urea application. Because variables analyzed were too many for PCA, some variable were selected for PCA based on ANOVA results. The first principal component (PC1) explained 47.5% of the variance and the second principal component (PC2) explained 22.4% of the variance, which explained 69.9% of the total variation. The PIES, soil EC, and fresh and dry biomass showed positive loadings on PC1 while nutrient concentrations in stem extract and stem water content had negative loadings on PC1. Stem EC and pH, proline, and stem diameter were major contributors to PC2 (Fig. 4).

The two principal components could separate different amount of urea fertilized groups and PC1 contributed most significantly to the separation of high and low fertilized pepper plants. Pepper plants with high urea fertilizer showed high biomass, shoot height, soil EC, and chlorophyll content. The PCA is a statistical tool to assess associations of traits and discriminate plants with different growth characteristics under various environmental conditions affecting plant growth^42^. The PCA clearly showed that PIES was related to pepper growth under different urea applications and PIES can be used to evaluate plant growth under different growth conditions.

## Conclusions

PIES can be used as an indicator to evaluate the nutrient absorption capacity, physiological activity, and growth status of plants by measuring the movement of water and nutrients of plant stem. High amount of urea application resulted in higher PIES values and better growth for pepper plants as compared to low and control urea applications. The study demonstrated that the PIES reflected enhanced pepper plant biomass by fertilizer application, which was not identified by destructively measured growth parameters such as nutrient contents in plant, chlorophyll and proline contents. The PIES can be measured non-destructively and continuously during the period of plant growth. Therefore, the PIES can be used to evaluate and continuously monitoring plant growth under various environmental conditions.

## Supplementary Information

Supplementary Information.

## Data Availability

The data that support the findings of this study are available from the corresponding author, [J.H. Park], upon reasonable request.
