# Supplementary material for: Monitoring of plant-induced electrical signal of pepper plants (Capsicum annuum L.) according to urea fertilizer application
Source: Sci Rep. 2023 Jan 6;13:291. doi: 10.1038/s41598-022-26687-w (PMC9822957; doi:10.1038/s41598-022-26687-w)

**Monitoring of plant-induced electrical signal of pepper plants (*Capsicum annuum* L.) according to urea fertilizer application**

Han Na Kim, Yeong Ju Seok, Gyung Min Park, Govind Vyavahare, Jin Hee Park*

Supplementary Table 1. Characteristics of soils used for greenhouse and field experiments

| Sample | Soil texture | pH | EC (μS/cm) | SOM (%) | NH_4_^+^-N (mg/kg) | NO_3_^-^-N (mg/kg) | Available P (mg/kg) | CEC (cmol_c_/kg) |
| --- | --- | --- | --- | --- | --- | --- | --- | --- |
| Greenhouse | Sandy loam | 6.5 | 55.3 | 1.7 | 2.2 | 420 | 69.5 | 9.7 |
| Field | Loam | 6.5 | 228 | 1.0 | 7.8 | 298 | 20.7 | 10.8 |

Supplementary Figure 1. Schematic diagram and photo of the sensor inserted in the stem of pepper


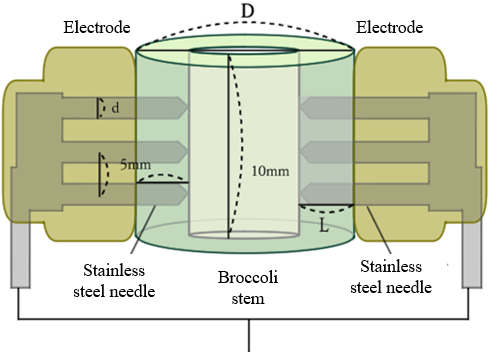

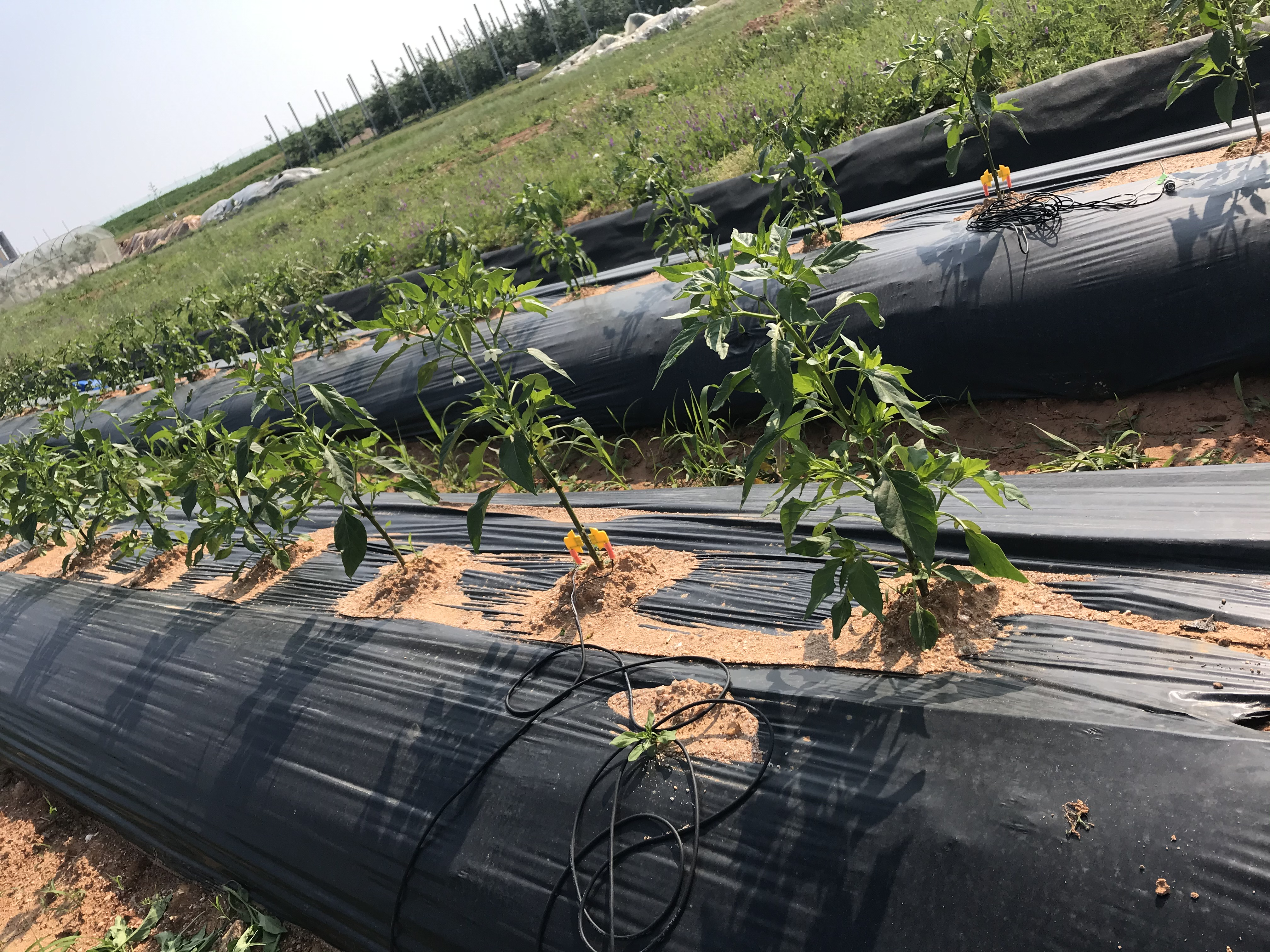

Supplement: Supplementary file 1 — Supplementary Information. [file 41598_2022_26687_MOESM1_ESM.docx]
